# PathwayBench: a multi-criterion benchmark of pseudobulk pathway activity scoring methods reveals rank-window competition as a mechanism of biological signal loss in single-cell RNA-seq

**DOI:** 10.21203/rs.3.rs-10271634/v1

**Published:** 2026-07-10

**Authors:** Fadhl M. Alakwaa, Ahmed Elbaz, Sara Al Azzam, Tasnim Alshebani

**Affiliations:** University of Michigan; American University in Cairo; Jordan University of Science and Technology; Wayne State University

**Keywords:** Single-cell RNA-seq, Pathway activity scoring, Benchmarking, Pseudobulk, ssGSEA, GSVA, AUCell, UCell, Chronic kidney disease, Rank-window competition

## Abstract

**Background:**

Pathway activity scoring is a foundational step in single-cell RNA-seq (scRNA-seq) analysis, yet method choice is rarely guided by systematic, multi-criterion evidence in the pseudobulk case-control regime that now dominates applied single-cell disease studies. Prior single-cell pathway-scoring benchmarks have focused on perturbation ground truth at cell-level resolution, leaving the donor-level pseudobulk setting — and the question of why methods disagree — largely unaddressed.

**Results:**

We present PathwayBench, a benchmark comparing five widely used pathway scoring methods (ssGSEA, GSVA, z-score, AUCell, UCell) across eight pseudobulked scRNA-seq case-control datasets spanning five tissues (brain, heart, kidney, lung, blood) and 682 donors. Methods are evaluated against five criteria covering biological relevance (direction accuracy, AUROC, effect size) and four robustness axes (aggregation, outlier, normalization, sample-size stability). We identify rank-window competition, a mechanism by which rank-based methods (AUCell, UCell) lose biological signal — and in extreme cases invert it — when non-pathway competitor genes saturate the top-rank scoring window. Controlled simulations demonstrate sign inversion in 40% of replicates under high competitor burden, and real-data confirmation comes from extracellular matrix (ECM) remodeling in chronic kidney disease, where rank-based methods produce wrong-direction effects. Discretizing per-criterion performance as Good/Intermediate/Poor reveals that no single method satisfies all five criteria, with GSVA and UCell each meeting four along different axes.

**Conclusions:**

Rank-window competition provides a mechanistic explanation for systematic divergence between magnitude-aware and rank-based pathway scoring methods on scRNA-seq data and should guide method selection, particularly for fibrotic, inflammatory, or otherwise broadly remodeled transcriptomes. PathwayBench is delivered as a versioned, extensible benchmark with per-dataset scores, an interactive advisor application, and complete reproducibility infrastructure to support evidence-based method selection and community extension.

## Background

Single-cell RNA sequencing (scRNA-seq) has shifted analytical attention from individual genes toward modules of co-regulated genes that summarize coordinated biology. Pathway activity scoring transforms the high-dimensional, sparse single-cell expression matrix into compact, interpretable scores that downstream analyses use to compare conditions, define cell states, and prioritize mechanism. Five methods dominate this step in practice: single-sample Gene Set Enrichment Analysis (ssGSEA) [1], Gene Set Variation Analysis (GSVA) [2], the standardized mean (z-score) [3], AUCell [4], and UCell [5].

These methods fall naturally into two families. Magnitude-aware methods (ssGSEA, GSVA, z-score) compute a continuous score from the relative expression magnitudes of pathway genes versus background. Rank-based methods (AUCell, UCell) compute a score from where pathway genes fall within a fixed top-rank window of the expression-sorted gene list. The two families are usually presented as alternative implementations of the same idea and treated as roughly interchangeable in practice — the choice is often dictated by a tutorial, a vignette, or a single influential paper rather than by systematic evidence.

Prior single-cell pathway-scoring benchmarks have focused on different regimes and questions. Holland et al. [34] benchmarked transcription factor and pathway methods on single-cell perturbation data with ground truth from CRISPR/CRISPRi experiments, and showed that performance depends more on gene-set choice than on scoring statistic. Zhang et al. [36] compared seven pathway activity methods — including AUCell, GSVA, ssGSEA, z-score, Pagoda2, Vision, and PLAGE — on single-cell clustering robustness and normalization sensitivity. Neither benchmark addresses the pseudobulk case-control regime that now dominates applied single-cell disease studies [11, 12], and neither provides a mechanistic account of why magnitude-aware and rank-based methods diverge. More general benchmarks have advanced related steps in single-cell analysis — clustering [6], integration [7], trajectory inference [8], and differential expression [9, 10] — but pathway scoring in the pseudobulk regime has received less systematic attention.

Two gaps motivate this work. First, biological relevance — usually operationalized as direction accuracy against literature ground truth — is a necessary but insufficient bar; a useful score must also be stable across reasonable variation in normalization, aggregation, sample size, and outliers. Second, when methods do disagree, the field lacks a mechanistic account of why: practitioners observe that AUCell and ssGSEA give different answers on the same data, but the algorithmic reason is rarely articulated.

PathwayBench addresses both gaps. We curate eight pseudobulked scRNA-seq case-control datasets from CZ CELLxGENE Discover [35] and the Kidney Precision Medicine Project (KPMP) [25], spanning Alzheimer’s disease, Parkinson’s disease, COVID-19 (lung and blood), idiopathic pulmonary fibrosis, dilated cardiomyopathy, systemic lupus erythematosus, and chronic kidney disease (CKD) across five tissues. We score each dataset with five methods, evaluate each method on five criteria, and classify per-criterion performance using a Good/Intermediate/Poor framework. We then identify rank-window competition as a mechanistic explanation for the systematic divergence between magnitude-aware and rank-based methods, demonstrate it in controlled simulations, and confirm it in a clinically meaningful real-data setting (ECM remodeling in CKD). The benchmark, per-dataset scores, evaluation code, an interactive advisor application, and complete reproducibility infrastructure are released as a versioned scaffold for the community to extend.

## Results

### Benchmark design

**Figure 1:**
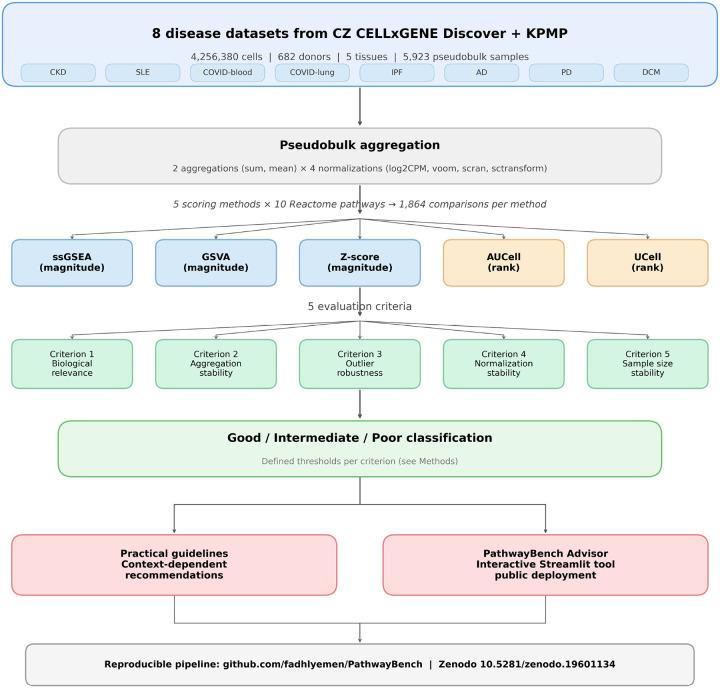
PathwayBench design overview. Eight pseudobulked scRNA-seq case-control datasets (4,256,380 cells, 682 donors, 5 tissues, 5,923 pseudobulk samples) from CZ CELLxGENE Discover and KPMP are scored by five pathway activity methods across 10 Reactome pathways (1,864 comparisons per method after ground-truth curation) and evaluated on five criteria. Per-criterion performance is classified as Good, Intermediate, or Poor against fixed thresholds. Results inform practical guidelines, a public interactive advisor, and a reproducible pipeline.

PathwayBench evaluates five pathway activity scoring methods (ssGSEA, GSVA, z-score, AUCell, UCell) across eight pseudobulked scRNA-seq case-control datasets comprising 4,256,380 cells from 682 donors across five tissues — brain (ad_brain, pd_brain), blood (covid_blood, sle_blood), kidney (ckd_kidney), lung (covid_lung, ipf_lung), and heart (dcm_heart) — yielding 5,923 pseudobulk samples and 1,864 pathway-level comparisons per method across 10 Reactome pathways (Fig. 1; Additional file 1: Table S1). Each method was evaluated against five criteria: biological relevance, aggregation stability, outlier sensitivity, normalization stability, and sample-size stability. All counts were aggregated by uniform pseudobulk summation followed by log_2_ counts-per-million (CPM) transformation, and all summary statistics were computed as the mean of per-dataset means to avoid datasets with more pathways dominating the comparison. Method performance per criterion was discretized into Good, Intermediate, or Poor using fixed thresholds (Methods).

### Biological relevance varies modestly across methods

**Figure 2:**
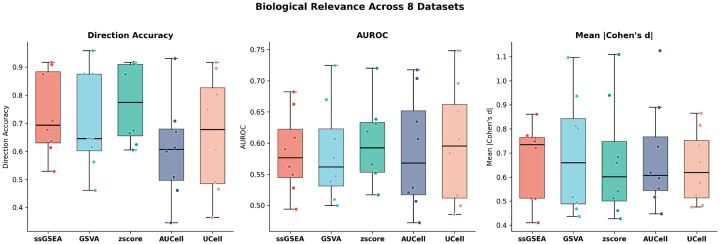
Biological relevance across eight datasets. Per-dataset values (points) and distributions (boxplots) for direction accuracy (left), AUROC (center), and mean absolute Cohen’s d (right) for each of the five scoring methods. Each box summarizes the eight per-dataset means.

All five methods detect literature-supported case-control direction at well-above-chance rates, but with a meaningful sensitivity gradient (Fig. 2; Additional file 1: Table S2). Direction accuracy ranged from 0.605 (AUCell) to 0.773 (z-score), with ssGSEA (0.733), GSVA (0.705), and UCell (0.662) intermediate. Discrimination measured by AUROC was tightly clustered (0.584–0.600) across all five methods. Mean absolute Cohen’s d, which captures effect-size magnitude rather than direction, ranked GSVA highest (0.695), followed by AUCell (0.684), z-score (0.667), ssGSEA (0.663), and UCell (0.642). Per-dataset variation was substantial (Additional file 1: Table S2) — for example, AUCell achieved direction accuracy of 0.931 on sle_blood but only 0.346 on ckd_kidney — pointing to a dataset-dependent factor we examine next.

### Rank-window competition explains systematic method divergence

**Figure 3:**
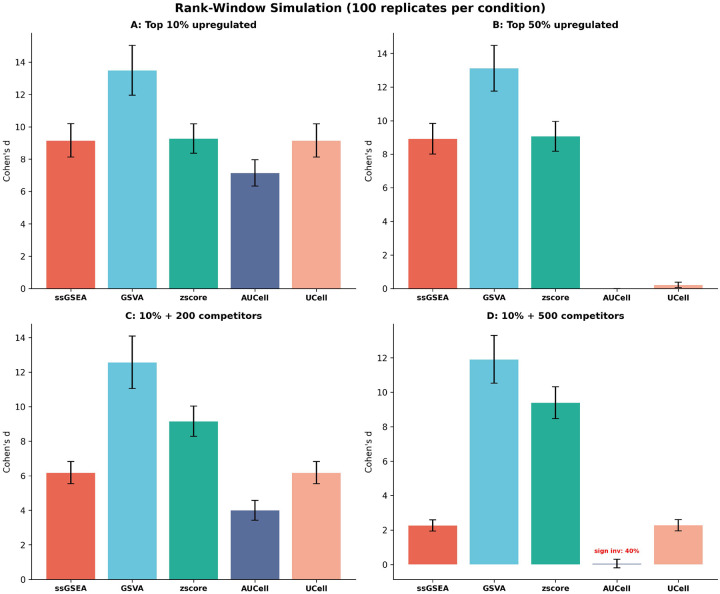
Rank-window competition simulation. Mean Cohen’s d (bar) and standard deviation (error bar) across 100 replicates per condition for each method under four scenarios of increasing competitor-gene burden. (A) Top 10% of pathway genes upregulated, no competitors. (B) Top 50% of pathway genes upregulated, no competitors. (C) Top 10% with 200 unrelated competitor genes. (D) Top 10% with 500 unrelated competitor genes; AUCell exhibits sign inversion in 40% of replicates (red label).

To isolate the mechanism behind the dataset-dependent gap between magnitude-aware and rank-based scoring, we constructed a controlled simulation with four scenarios of increasing competitor-gene burden, each with 100 replicates (Fig. 3). In Scenario A — the cleanest case, a focal 10% of pathway genes upregulated with no background competition — all five methods recovered a strong signal: GSVA produced the largest effect (Cohen’s d = 13.49 ± 1.54), followed by z-score (9.28 ± 0.91), ssGSEA (9.16 ± 1.04), UCell (9.15 ± 1.04), and AUCell (7.14 ± 0.81). In Scenario B, where 50% of pathway genes were upregulated, the magnitude-aware methods retained their effect (GSVA 13.12 ± 1.36, z-score 9.07 ± 0.89, ssGSEA 8.93 ± 0.91), but the rank-based methods collapsed: UCell fell to 0.22 ± 0.16 and AUCell to 0.00 ± 0.00. With a focal 10% signal embedded in 200 unrelated competitor genes (Scenario C), AUCell dropped to 3.99 ± 0.57 and ssGSEA also began to attenuate (6.18 ± 0.65, down from 9.16 in Scenario A, closely tracking UCell at 6.19 ± 0.65), while z-score (9.17 ± 0.88) and GSVA (12.57 ± 1.52) remained largely intact. With 500 competitor genes (Scenario D), AUCell collapsed to 0.05 ± 0.25 and exhibited sign inversion in 40% of replicates, while UCell (2.27 ± 0.32) and ssGSEA (2.26 ± 0.32) attenuated but kept the correct sign; only z-score (9.40 ± 0.93) and GSVA (11.91 ± 1.38) sustained the signal. The pattern is consistent with rank-window competition: when many genes — pathway or otherwise — saturate the top-rank window used by AUCell and UCell, the within-window position of true pathway genes becomes uninformative, collapsing the score and, at the extreme, flipping its sign.

### Rank-window competition operates in real disease data

**Figure 4:**
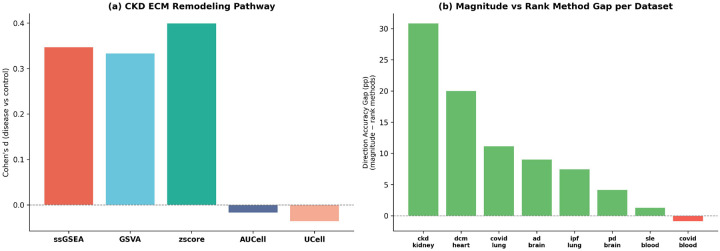
Rank-window competition in real data. (a) Per-method Cohen’s d (disease vs. control) for the ECM remodeling pathway in CKD, averaged across three pseudobulk normalization schemes (log2CPM, scran, sctransform). Magnitude-aware methods (ssGSEA, GSVA, z-score) detect the expected upregulation under all three normalizations; rank-based methods (AUCell, UCell) yield near-zero or wrong-direction effects under all three. (b) Direction-accuracy gap (magnitude − rank, percentage points) for each of the eight datasets, ordered from largest to smallest gap.

The same mechanism is detectable in a clinically relevant setting. In CKD, ECM remodeling is a hallmark of progressive fibrosis and is expected to be upregulated in disease versus control [27]. For the ECM remodeling pathway scored under three pseudobulk normalization schemes (log2CPM, scran, sctransform) as a robustness check, magnitude-aware methods consistently identified upregulation across all three normalizations (mean Cohen’s d: z-score = 0.399, ssGSEA = 0.347, GSVA = 0.334), whereas rank-based methods produced near-zero or wrong-direction effects across all three (mean Cohen’s d: AUCell = − 0.017, UCell = − 0.036) (Fig. 4a; Additional file 1: Table S5). The magnitude-versus-rank direction-accuracy gap, computed across all CKD ground-truth pathways under the manuscript’s default log2CPM normalization, was largest in ckd_kidney (+ 30.8 percentage points), followed by dcm_heart (+ 20.0 pp), covid_lung (+ 11.2 pp), ad_brain (+ 9.1 pp), ipf_lung (+ 7.4 pp), pd_brain (+ 4.1 pp), sle_blood (+ 1.3 pp), and covid_blood (− 0.9 pp) (Fig. 4b; Additional file 1: Table S2). The largest gaps occur in tissues — kidney and heart — whose disease signatures are dominated by broad upregulation of structural and inflammatory programs that flood the top-rank window.

### No single method satisfies all five criteria

**Figure 5:**
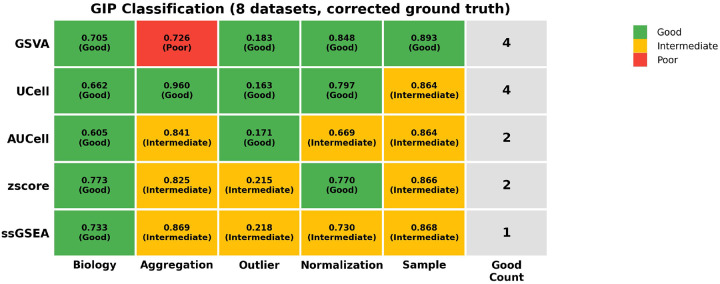
Good/Intermediate/Poor classification across five criteria. Per-method per-criterion mean values (numerals) discretized into Good (green), Intermediate (yellow), or Poor (red) using fixed thresholds (Methods). The rightmost column shows the count of Good cells per method.

Discretizing each method’s per-criterion mean against fixed thresholds (Fig. 5; Additional file 1: Table S3) yields a Good/Intermediate/Poor classification with no overall winner. GSVA achieved Good on four of five criteria — biology (0.705), outlier sensitivity (0.183, lower is better), normalization stability (0.848), and sample-size stability (0.893) — but Poor on aggregation stability (0.726). UCell also achieved Good on four criteria, including the highest aggregation stability of any method (0.960), with Intermediate on sample-size stability (0.864). Z-score and AUCell each met two Good criteria; ssGSEA met one. UCell and GSVA emerge as the most balanced choices when both biology and stability matter, but for any specific analysis the relevant subset of criteria — and therefore the recommended method — depends on the experimental design.

### Sensitivity and robustness trade off across the method panel

**Figure 6:**
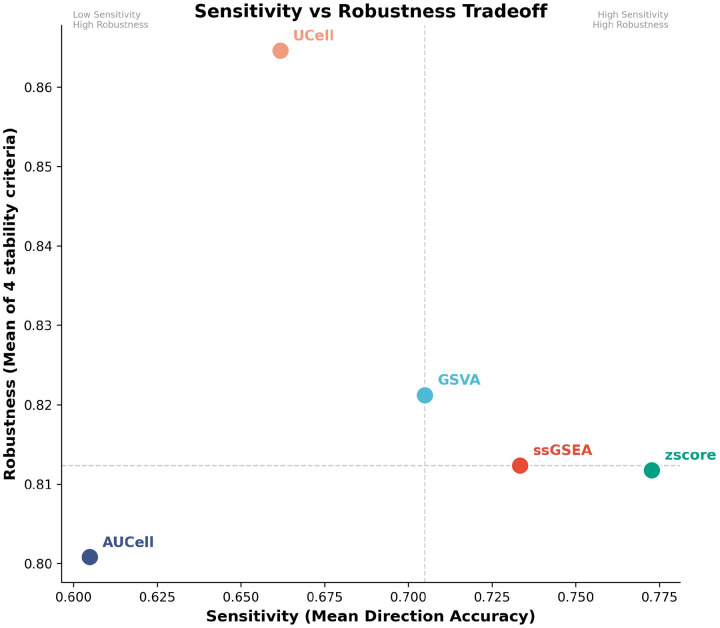
Sensitivity–robustness tradeoff. Mean direction accuracy (x-axis, sensitivity) versus the mean of the four robustness criteria — aggregation stability, outlier robustness (1 − outlier sensitivity), normalization stability, and sample-size stability — (y-axis). Dashed lines mark median sensitivity (0.705) and median robustness (0.812).

Plotting mean direction accuracy (sensitivity) against the mean of the four robustness criteria (Fig. 6) places the methods in four distinct positions. UCell occupies the high-robustness, moderate-sensitivity quadrant (0.662, 0.865); z-score occupies the high-sensitivity, median-robustness position (0.773, 0.812); GSVA sits centrally (0.705, 0.821); ssGSEA tracks z-score on robustness (0.733, 0.812); AUCell anchors the low/low corner (0.605, 0.801). The median sensitivity (0.705) and median robustness (0.812) divide the plane into selection regions that map directly onto practitioner priorities.

### An interactive advisor and decision tree operationalize method selection

**Figure 7:**
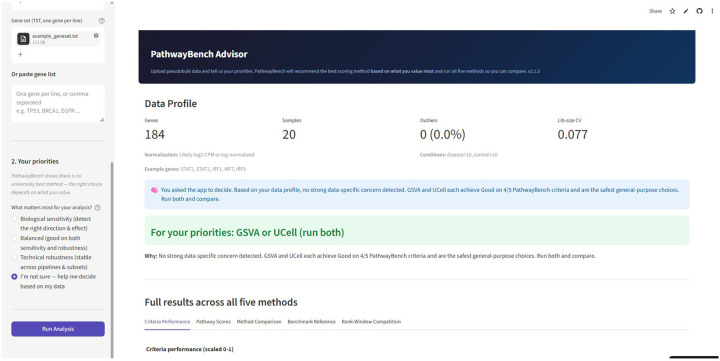
PathwayBench Advisor application. Screenshot of the public Streamlit application (pathwaybench-xrdxczzdpeahvcinxwbrcq.streamlit.app). Users upload a pseudobulk gene set and select an analysis priority; the advisor profiles the data, detects potential concerns (outliers, library-size variation, normalization), and returns a context-dependent method recommendation with supporting evidence from the benchmark. Shown: the “I’m not sure” escape hatch, which triggers data-driven triage and recommends GSVA or UCell (both 4/5 Good criteria) as general-purpose defaults.

**Figure 8:**
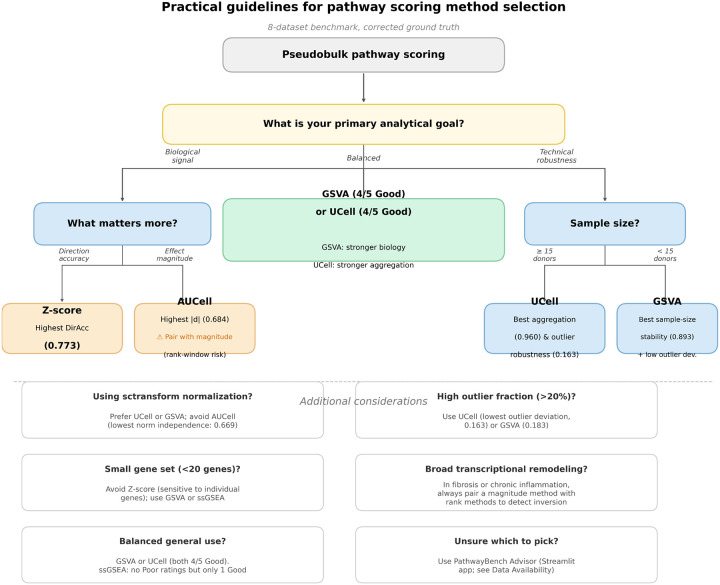
Practical guidelines for pathway scoring method selection. Decision tree guiding method choice based on the user’s primary analytical goal (biological signal, balanced, or technical robustness). Terminal nodes show the recommended method with the deciding criterion value from the benchmark. Z-score achieves the highest direction accuracy (0.773); AUCell the highest effect size (|d| = 0.684) but carries rank-window risk; GSVA and UCell (both 4/5 Good) serve as balanced defaults. Lower panel lists additional considerations for specific data characteristics, including normalization choice, outlier burden, gene-set size, and broad transcriptional remodeling.

Because no method dominates and the right choice depends on which criteria matter most for a given study, we built a public Streamlit application — PathwayBench Advisor — that lets users specify their priorities (e.g., maximum sensitivity vs. cross-normalization stability) and returns a ranked recommendation with the underlying per-criterion evidence (Fig. 7). The same logic is summarized as a printable decision tree (Fig. 8): users prioritizing biological signal choose z-score for direction accuracy (0.773) or AUCell for effect-size magnitude (0.684, paired with a magnitude method to guard against rank-window risk); users seeking a balanced default choose GSVA or UCell (both 4/5 Good); users prioritizing technical robustness choose between UCell for larger studies (≥ 15 donors; aggregation stability 0.960) and GSVA for smaller ones (sample-size stability 0.893). An additional-considerations panel addresses normalization choice, outlier burden, small gene sets, and broad transcriptional remodeling.

## Discussion

PathwayBench yields three findings of practical and methodological consequence. First, rank-window competition is a coherent mechanistic explanation for the systematic divergence between magnitude-aware and rank-based pathway scoring methods. The simulation isolates the mechanism — at high competitor-gene burden, the top-rank window used by AUCell and UCell saturates and the score loses (or, at the extreme, inverts) its biological direction — and the CKD ECM analysis confirms that the same mechanism operates in real data, with the largest magnitude-versus-rank direction-accuracy gaps appearing in tissues whose disease signature involves broad upregulation of dense functional programs. Notably, susceptibility to this mechanism is a matter of degree rather than a clean two-family split: although ssGSEA is magnitude-based by construction, under strong competitor burden it attenuates more like a rank-based method than like the genuinely competition-resistant z-score and GSVA, occupying an intermediate position. Practitioners scoring fibrotic, inflammatory, or otherwise diffuse signatures with rank-based methods — or with ssGSEA — should expect attenuation, and in extreme cases sign inversion.

Second, the Good/Intermediate/Poor classification reframes the long-running debate over the “best” pathway scoring method as a question of analysis priorities rather than a single ranking. GSVA and UCell each cleared four of five criteria, but along entirely different axes — GSVA’s Poor aggregation-stability cell (0.726) reflects sensitivity to how counts are aggregated to the donor level, while UCell’s Intermediate sample-size cell (0.864) reflects mild instability under subsampling. Z-score’s high direction accuracy (0.773) is offset by intermediate scores on three of the four robustness criteria. AUCell pays for its rank-based design with the lowest sensitivity (0.605) and the lowest normalization stability (0.669). The right method depends on which trade-off the analysis can absorb.

Third, PathwayBench is a scaffold rather than a closed verdict. The benchmark is reproducible from raw data through final figures using the released code, the per-dataset scores are available for re-analysis, and the framework is structured so that new methods (decoupleR [13], Hotspot [14], PAGODA [15]), new datasets, and new criteria can be added without re-architecting the pipeline. The PathwayBench Advisor application makes the per-criterion evidence directly inspectable, and the decision tree provides a printable summary for routine analysis planning.

### Limitations

Several limitations bound the present results. The eight datasets, while spanning five tissues and a range of disease etiologies, are not exhaustive — pathways and tissues with very different signature density may behave differently. All scoring used uniform sum-aggregation followed by log_2_ CPM normalization; methods with intrinsic normalization-invariance (AUCell, UCell) and methods that benefit from explicit normalization (ssGSEA, GSVA, z-score) were therefore evaluated on the same input, which is the defensible benchmark choice but is not the only reasonable one. The number of literature-supported ground-truth pathways varies across datasets, and per-dataset comparison counts are reported in the released metrics file. Finally, ground-truth pathway sets evolve as disease biology is refined, and the benchmark should be re-run as those sets are updated.

## Conclusions

PathwayBench provides a systematic, multi-criterion comparison of pathway activity scoring methods in the pseudobulk scRNA-seq case-control regime. Our three principal contributions are: (1) a mechanistic account — rank-window competition — of why rank-based and magnitude-aware methods diverge, supported by controlled simulations and confirmed in real disease data; (2) a five-criterion evaluation framework spanning biological relevance and four robustness axes, which shows that no single method satisfies all criteria and reframes method selection as a question of analysis priorities; and (3) an extensible, reproducible benchmark scaffold — including per-dataset scores, an interactive advisor, and a decision tree — that the community can extend with new methods, datasets, and criteria. Practitioners analyzing fibrotic, inflammatory, or otherwise broadly remodeled transcriptomes should prefer magnitude-aware methods or explicitly check for rank-window competition before trusting rank-based scores.

## Methods

### Datasets and pseudobulk aggregation

Eight publicly available scRNA-seq case-control datasets were included: ad_brain (Alzheimer’s disease, prefrontal cortex) [17], pd_brain (Parkinson’s disease, midbrain) [18], ckd_kidney (CKD) [24, 25], dcm_heart (dilated cardiomyopathy) [23], covid_lung and ipf_lung (COVID-19 and idiopathic pulmonary fibrosis) [19, 20], and covid_blood and sle_blood (COVID-19 and systemic lupus erythematosus, peripheral blood) [21, 22]. Datasets were obtained from CZ CELLxGENE Discover [35] and the KPMP [25]. Counts were pseudobulked to the donor × cell-type level by uniform summation, then transformed to log_2_(counts per million + 1) for input to all scoring methods.

### Pathway scoring

Five methods were evaluated using their reference implementations: ssGSEA [1] and GSVA [2] via the GSVA R package; the standardized mean (z-score) [3] as the per-pathway mean of per-gene z-scores across donors; AUCell [4] via the AUCell R package with default top-rank window settings; and UCell [5] via the UCell R package. All methods were applied to the same log_2_ CPM input matrices.

### Ground-truth pathway sets

For each dataset, literature-supported case-control pathway gene sets were drawn from Reactome [16] — retrieved via the MSigDB C2:CP:REACTOME collection, with a Reactome-API fallback — and each pathway was labeled with an expected direction (up- or downregulated in disease). Expected directions were assigned from a review of the published disease literature [17–25]. For the CKD ECM remodeling robustness analysis (Fig. 4a; Additional file 1: Table S5), the CKD ground-truth ECM remodeling pathway was scored under three pseudobulk normalization schemes (log2CPM, scran, sctransform) [26, 27] to test whether the rank-window competition signal persists across normalization choices.

### Biological relevance metrics

Direction accuracy was computed per pathway as the fraction of donor-level case-vs-control comparisons in which the score moved in the expected direction; AUROC was computed for case-vs-control discrimination on the per-donor pathway scores; |Cohen’s d| was computed as the absolute standardized mean difference between case and control. Per-dataset means were averaged across the eight datasets to produce the values reported in Fig. 2.

### Robustness criteria

Aggregation stability was the Spearman correlation of per-pathway scores across alternative donor pseudobulk aggregation strategies (sum, mean, median). Outlier sensitivity was the mean absolute change in score after a single-donor outlier was injected (lower is better); the corresponding outlier robustness used in Fig. 6 is defined as 1 − outlier sensitivity. Normalization stability was the Spearman correlation of scores computed under alternative normalization schemes (log_2_ CPM, log_2_ TPM, no normalization). Sample-size stability was the Spearman correlation of scores under random subsampling of donors.

### Good/Intermediate/Poor thresholds

Per-criterion thresholds were fixed in advance: Biology — direction accuracy > 0.45 (Good), > 0.30 (Intermediate), ≤ 0.30 (Poor); Aggregation stability — ≥0.90 (Good), ≥ 0.75 (Intermediate), < 0.75 (Poor); Outlier sensitivity — <0.20 (Good), < 0.27 (Intermediate), ≥ 0.27 (Poor); Normalization stability — ≥0.75 (Good), ≥ 0.65 (Intermediate), < 0.65 (Poor); Sample-size stability — ≥0.88 (Good), ≥ 0.84 (Intermediate), < 0.84 (Poor).

### Rank-window competition simulation

Synthetic count matrices were generated with 100 replicates per scenario, 200 donors per replicate (100 case, 100 control), and 1,000 baseline genes. Four scenarios varied the upregulated fraction of the focal pathway and the number of unrelated competitor genes co-upregulated in cases: A (focal 10%, no competitors), B (focal 50%, no competitors), C (focal 10% + 200 competitors), D (focal 10% + 500 competitors). Per-scenario per-method Cohen’s d was computed between case and control donor scores; sign inversion rate was the fraction of replicates in which the case-vs-control sign flipped relative to the simulated direction.

### Aggregation across datasets

All summary statistics reported in the main text are means of per-dataset values across the eight datasets. This avoids datasets with more pathways dominating the comparison.

### Software and reproducibility

Analyses were performed in R 4.1.0 on RHEL 8.10 within a managed conda environment. Single-cell processing relied on Seurat [28] and SCANPY [29]. Differential expression and statistics relied on edgeR [30], DESeq2 [31], and limma [32]. Scripts, environment specification, per-dataset metrics, and figure-generation code are in the public repository. Code development used Claude Code (Anthropic) and OpenAI Codex; all outputs were independently reviewed and verified by the authors.

## Supplementary Material

Supplementary Files

This is a list of supplementary files associated with this preprint. Click to download.

- PathwayBenchSupplementaryTableswithS5.docx

## Data Availability

All eight datasets are available from CZ CELLxGENE Discover (https://cellxgene.cziscience.com) and the Kidney Precision Medicine Project (https://kpmp.org; RRID:SCR_026845) under their respective access terms. The PathwayBench code, ground-truth pathway sets, evaluation scripts, and figure-generation scripts are available at https://github.com/FADHLyemen/PathwayBench and archived at Zenodo (https://doi.org/10.5281/zenodo.19415048; release v3.1). The supporting data — pseudobulk expression matrices, per-dataset pathway scores, evaluation outputs, and rank-window simulation data — are deposited at Zenodo (https://doi.org/10.5281/zenodo.21122230). To reproduce the analysis, clone the repository at tag v3.1 and unpack the data archive into the repository root. The interactive PathwayBench Advisor application is available at https://pathwaybench-xrdxczzdpeahvcinxwbrcq.streamlit.app.

## Abbreviations

AUROC: Area under the receiver operating characteristic curve
CKD: Chronic kidney disease
CPM: Counts per million
DCM: Dilated cardiomyopathy
ECM: Extracellular matrix
GSVA: Gene Set Variation Analysis
IPF: Idiopathic pulmonary fibrosis
KPMP: Kidney Precision Medicine Project
scRNA-seq: Single-cell RNA sequencing
SLE: Systemic lupus erythematosus
ssGSEA: Single-sample Gene Set Enrichment Analysis
TF: Transcription factor
